# Sound stimulation using the individual's heart rate to improve the stability and homeostasis of the autonomic nervous system

**DOI:** 10.14814/phy2.15816

**Published:** 2023-09-19

**Authors:** Daechang Kim, Nahyeon Kim, Younju Lee, Sungmin Kim, Jiyean Kwon

**Affiliations:** ^1^ Department of Medical Biotechnology Dongguk University Gyeonggi‐do Korea; ^2^ Department of Medical Device and Healthcare Dongguk University Seoul Korea

**Keywords:** autonomic nervous system, homeostasis, personalization, sound stimulation, stability

## Abstract

**Objectives:**

In this study, we explain the role of enhancing the stability and homeostasis of the autonomic nervous system (ANS) by proposing the average heart rate sound resonance (aHRSR), a sound stimulation to prevent imbalance of ANS due to dynamic movement. The effect of aHRSR on ANS was analyzed through the time and frequency domain of heart rate variability (HRV) using the photoplethysmogram data (PPG) of 22 participants (DUIRB‐202109‐12).

**Method:**

When the subjects performed dynamic movements that could cause changes in the ANS, HRV indicators using PPG data for 5 min before and after the movements were analyzed according to the presence or absence of aHRSR. The standard deviation of the NN intervals (SDNN), the square root of the mean squared differences of the NN intervals (RMSSD), low‐frequency band (LF), and high‐frequency band (HF), which represent sympathetic and parasympathetic nerve activity, were used as indicators, where SNDD and LF represent total ANS and sympathetic activity, while RMSSD and HF represent parasympathetic activity.

**Results:**

As the effects of aHRSR on dynamic movement, the recovery time of RR interval was advanced by about 15 s, SDNN increased from ([44.16 ± 13.11] to [47.85 ± 15.16]) ms, and RMSSD increased from ([23.73 ± 9.95] to [31.89 ± 12.48]) ms (*p* < 0.05), increasing the stability of the ANS and reducing instability. The effect of homeostasis of the ANS according to aHRSR is also shown in reducing the change rate of LF from (−13.83 to −8.83) %, and the rate of change of HF from (10.59 to 3.27) %.

**Conclusions:**

These results suggest that aHRSR can affect the cardiovascular system by assisting physiological movements that occur during dynamic movement.

## Graphical abstract

In this study, sound stimulation improves the stability and homeostasis of the autonomic nervous system by stimulating the vagus nerve in dynamic movement. Sound stimulation using an individual's heart rate affects blood pressure regulation and shows rapid recovery from rapid changes. In the future, it is expected to be used as a method of biofeedback to help individuals' health.

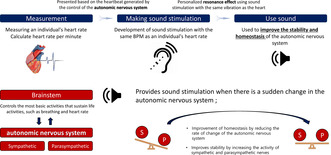

## 1 INTRODUCTION

The autonomic nervous system (ANS), composed of the sympathetic nervous system (SNS) and parasympathetic nervous system (PNS), is distributed in all organs in the body. The ANS plays a key role in controlling the functions of the body to cope with various internal and external environmental changes. In general, the SNS is involved in emergency functions. When the body is exposed to stress, the ANS suppresses the digestive system and increases heart activity to promote energy use. The PNS is involved in the protective function and acts as a contrast to the SNS (Beissner et al., 2013; Byung Rim Park, 2017). Some researchers compare the action of the SNS and PNS to a seesaw. To prevent the excessive activity of one nervous system, SNS and PNS use opposing forces to intelligently control each other. In other words, both nervous systems can effectively control and coordinate the function of all connected organs while maintaining in vivo homeostasis, depending on the situation (McCorry, 2007).

The sinus node in the heart induces the heart rate through spontaneous action potential (Monfredi et al., 2010). However, in the ganglion of the ANS existing around the sinus node, there are receptors that bind to acetylcholine and norepinephrine, which are neurotransmitters of the ANS, so that it is possible to control the action potential of the heart (Lee, 2012; Osterrieder et al., 1980). Accordingly, the heart responds sensitively to changes in the ANS, and the heart rate may change depending on the ANS. For this reason, heart analysis is being used as a tool to observe changes in the ANS, and this method is the heart rate variability (HRV). The HRV is an evaluation tool that can track the homeostasis control mechanism of the ANS in response to intrinsic and extrinsic environmental factors in real time. The ANS analysis using HRV is divided into time domain and frequency domain. Time domain analysis includes the standard deviation of normal‐to‐normal RR intervals (SDNN) and the root mean square of continuous NN interval differences (RMSSD), which are statistics calculated from heartbeat interval differences. Frequency domain analysis computes the periodic oscillatory information of a heartbeat at various frequencies through a fast Fourier transform. Generally, it is divided into a low‐frequency band (LF) of (0.05–0.15) Hz and a high‐frequency band (HF) of (0.15–0.4) Hz. Since the SDNN and LF are related to ANS activity, they are modulated according to changes in the SNS and PNS, while the RMSSD and HF are related to the activity of the vagus nerve, which belongs exclusively to the PNS (Bilchick & Berger, 2006; Rubinger et al., 2013; Zhou et al., 2012).

The electrocardiogram and photoplethysmogram (PPG), which can be measured in a noninvasive way, have made it easy to observe clinical changes in ANS in real time through HRV analysis. HRV analysis is used to observe changes in the balance and activity of the ANS in individual conditions and diseases, and to determine the effectiveness of medications and treatments (Sztajzel, 2004). As a result of these studies, deterioration of health and disease reduce the antagonistic function of the SNS and PNS, and finally cause imbalance of the ANS. Since this imbalance adversely affects the heart, which reacts sensitively to changes in ANS, it can ultimately have fatal effects on life, such as increased arrhythmia, and increased cardiovascular mortality (Buckley & Shivkumar, 2016; Carney et al., 2005; Kang & Choi, 2013; Kop et al., 2010; Lindgren et al., 1993; Podrid et al., 1984; Sgoifo et al., 1997). To prevent and treat these risks at present, severe patients use medical devices that stimulate the nervous system with electricity to activate functions, and take a drug that has a nerve‐blocking effect to prevent excess activity of the nervous system (Jongkees et al., 2018; Raj & Coffin, 2013).

However, the risks causing ANS imbalances not only occur in severe patients. The most common ANS imbalance in normal individuals without disease is vasovagal syncope and orthostatic dizziness. Because cardiovascular systems are gravity sensitive, a sudden change in body position, such as moving suddenly from a sitting position to standing position, results in a momentary drop in blood pressure, due to a decrease in blood flow to the heart. To protect against drop in blood pressure, the ANS immediately activates the SNS to normalize blood flow by increasing vessel constriction and the heart rate (Low & Singer, 2008), but external factors, such as excessive physical activity, mental stress, dehydration, and weather, can lead to abnormal feedback of the SNS, which constantly stimulates the PNS (Gupta & Lipsitz, 2007). This abnormal feedback causes rapid changes in the SNS and PNS, and breaks the balance. As a result, it causes vasodilation and decreased heart rate, and reduced blood flow to the brain can cause fainting and dizziness (Kim, 2011; Lee & Chung, 2006; Yoon & Kim, 2012). These risks do not have a lethal effect on life, but depending on the circumstances that occur, the risks can become very large; most importantly, there are few ways to manage and prevent sudden ANS imbalance.

Accordingly, this study proposes a new method to reduce the rapid imbalance of the ANS caused by postural changes that can commonly occur in normal circumstances to minimize the risks presented above, and finally suggests the role of the average heart rate sound resonance (aHRSR). Some studies have confirmed changes in the ANS when external sound stimulation is provided to individuals in a comfortable position (Lee et al., 2016; Paszkiel et al., 2020; Salamon et al., 2003; Thoma et al., 2013). However, the effects of sound stimulation on the rapid changes in the ANS that occur in non‐static postures could not be confirmed. Also, stimulation with vague criteria of sound will be difficult to use for treatment and prevention. To solve this problem, in this paper we develop a sound stimulation system with the same beats per minute (BPM) based on the participant's biosignal acquired through PPG, and propose a system that provides sound stimulation during sudden posture changes. Then, changes in the ANS due to sound stimulation are analyzed. Finally, it is confirmed that aHRSR can be applied to rapid changes in the ANS caused by dynamic movements, and can improve the stability and homeostasis of the ANS. It is expected that this technique can be used as a biofeedback training method and stimulation method that continuously manages the health of the individual's ANS as a personalized health management and mobile healthcare method.

## 2 MATERIALS AND METHODS

### 2.1 Recruitment method

This study was conducted with the approval of the Dongguk University Institutional Review Board (DUIRB‐202109‐12). The participants were a total of 22 people in their 20s and 30s with no history of cardiovascular diseases, such as arrhythmia or myocardial infarction, and of mental illness, such as depression or panic disorder. The gender ratio of participants was 12 males to 10 females. The average age was (26.36 ± 3.15) years. People over 40 years of age were excluded from this study, because they were more likely to have ANS abnormalities. All participants were informed of the purpose and methods of the study, and consent was obtained prior to data measurement.

### 2.2 Average heart rate sound resonance

Resonance is a phenomenon that allows large amplitudes and energies to be transferred, even though this is through the action of small forces at a certain frequency. The most representative theory is Schumann resonance. The most important property of Schumann resonance is that the electromagnetic field on earth and the physiological rhythm of the body can be synchronized (Alabdulgader, 2021; Alabdulgader et al., 2018; McCraty et al., 2017; Miller & Lonetree, 2013; Mitsutake et al., 2005). According to another paper on synchronization, physiological rhythms are changed similar to the frequency of certain repetitive movements (Blain et al., 2009; Daffertshofer et al., 2004), and visual and sound stimulation (Anishchenko et al., 2000; Bernardi et al., 2009). Based on this, this study suggests a hypothesis. The unique heart rate generated by the sinus node presents in the heart is (100–120) beat/min, but the action of the ANS determines the average heart rate for each individual. Typically, the average heart rate with ANS applied is (60–100) beat/min for adult standard. We suggest that this is the specific resonance frequency of the individual. In other words, we hypothesize that an external stimulation with the same frequency as an individual's heart rate could induce a specific change by transmitting large energy to the ANS by synchronizing with the individual's physiological rhythm, even with a small force.

**FIGURE 1:**
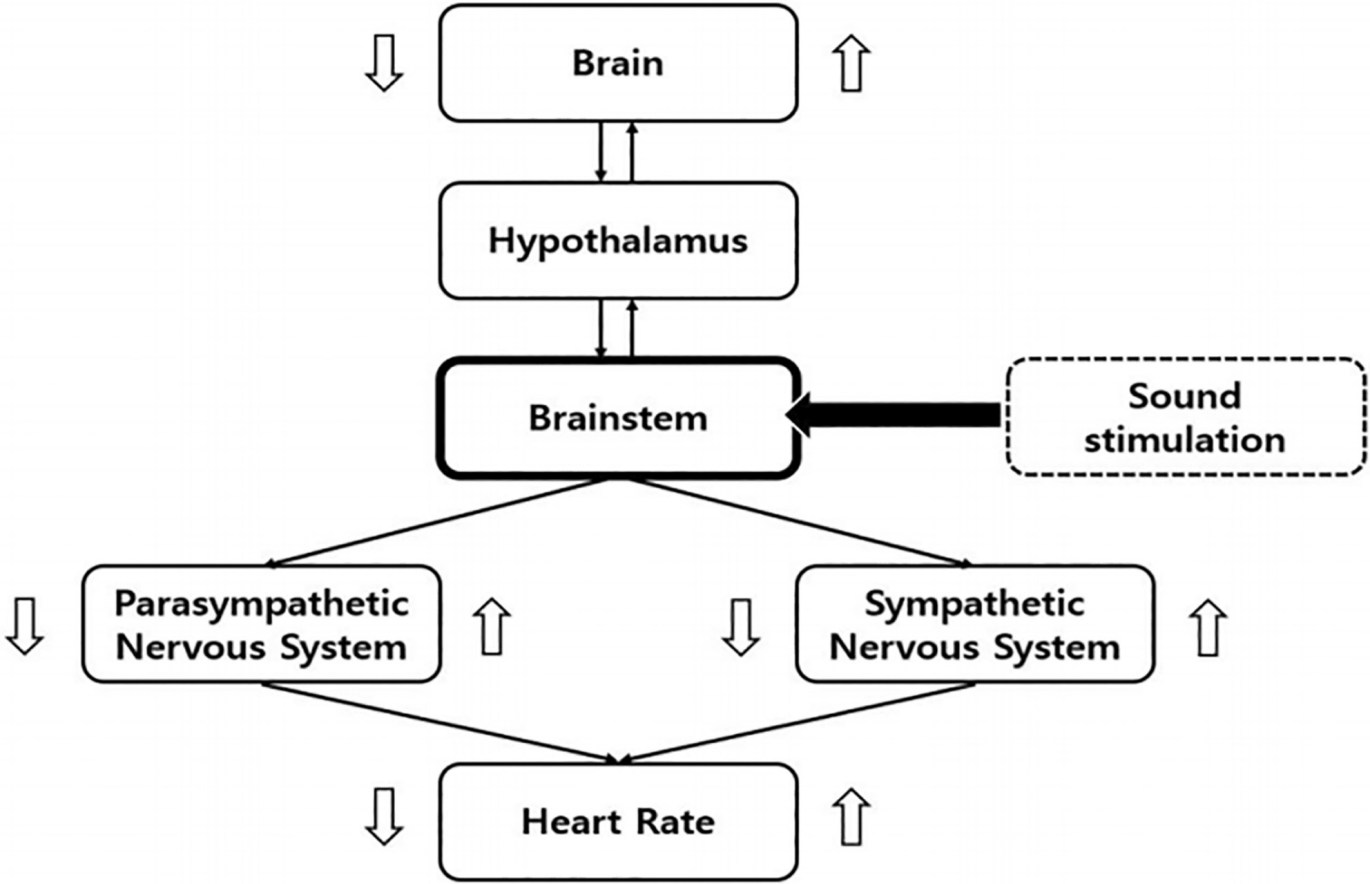
A simplified representation of the autonomic nervous system integration model and location of sound stimulation.

In this study, sound stimulation was chosen as external stimulation with resonance frequency. The rationale for this was based on the principle of brainstem auditory evoked potentials, which is used as a method of examining neurological diagnosis and polyvagal theory. The sound stimulation is transmitted to the eardrum and finally activates the brainstem to transmit auditory information (Picton et al., 1974). The brainstem has an integrated function of cardiovascular control, breathing control, and consciousness, and plays an important role in conduction, because all information relayed to the cerebral and cerebellum, or vice versa, must pass through the brainstem. In other words, activation of the brainstem using sound stimulation can affect the nervous system, and changes in ANS can be analyzed by HRV (Porges, 2003, 2011; Ray & Maunsell, 2011). For this reason, it was determined that the most effective stimulation to give resonance frequency was sound stimulation (shown in Figure 1).

We measured the average heart rate of the participants to obtain a resonance frequency and produced a 75 dB sound stimulation with the same tone frequency. The signal shape of the tone frequency follows the digital signal, and the tone duration is 0.2 s. The sound most similar to this type of sound stimulus is that made by the metronome. It was developed in the above form without using the analog tone frequency to minimize the acclimatization to sound stimuli, so that continuous stimulation can be given in the experimental procedure. It was named aHRSR.

### 2.3 Experimental procedure

**FIGURE 2:**
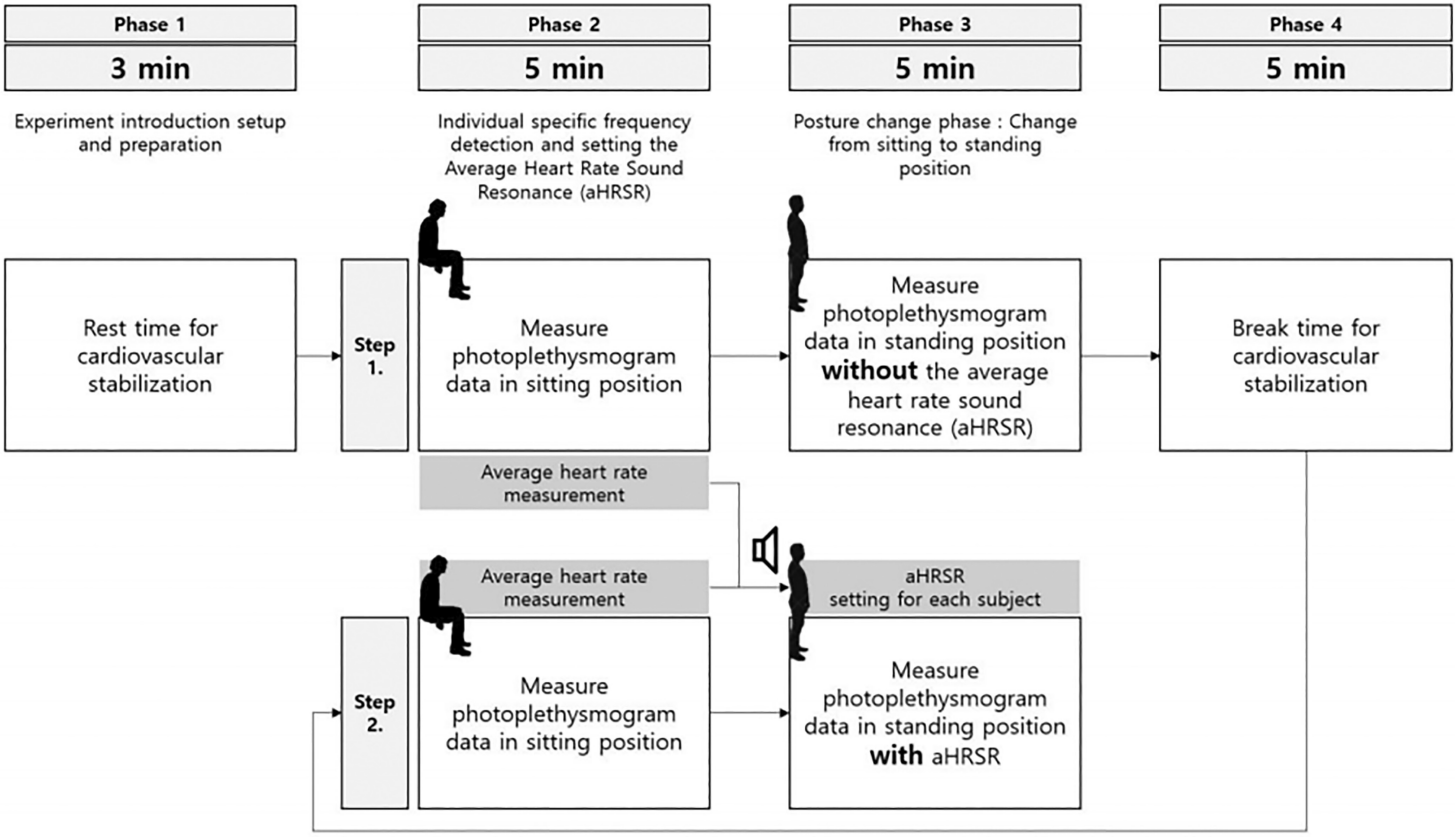
Experimental procedure. Test procedure for verifying the effects of the average heart rate sound resonance.

Figure 2 shows the procedure for measuring ANS and HRV to confirm the effect of aHRSR. All test environments were conducted under the same conditions in an external control environment except for auditory stimulation, and aHRSR was provided through a speaker. The sound provided a volume less than 80 dB, and the speaker was placed on the right side of the participant. To induce and observe sufficient changes in the cardiovascular system, the sitting and standing posture were set for 5 min each. PPG was measured using a Biopac System M36 and SS4LA pulse sensor. For the sensor value, sampling data of 1000 were used, and it was set to 5000 gain, 50 Hz low pass filter, and 60 Hz band‐stop filter, then clamped to the participant's index finger.

Phase represents the state of a participant. Phases 1 and 4 are the setup and initialization phases. Initialization is to stabilize ANS and HRV, which may have been altered by the initial and test movement. Phase 2 is PPG data measurement in the sitting position, while Phase 3 shows PPG data in the standing position. Each phase lasts 5 min, and the measurement time of the data is 5 min. Step is the presence or absence of aHRSR. Each participant had a total of 3 min rest while in the sitting position before the experiment. In Phase 1, the procedures for the total experiment were described and explained to the participant, and an SS4LA pulse sensor was attached. Next, the participant's PPG data were acquired for 5 min. After 5 min, the participant stood up immediately, and PPG data were measured for 5 min while maintaining the standing position. This process is Step 1 in Figure 2, and data were measured for a total of 10 min without aHRSR.

Participants were given a 5 min break after Step 1 to stabilize the changed ANS and HRV. The participants rested while sitting on a chair. Next, PPG was measured in the same way as in Step 1, and the average heart rate of the PPG data of Phase 2 measured in Step 1 and Step 2 was set as the BPM of aHRSR. Finally, in Phase 3 of Step 2, aHRSR was provided in line with the change in posture, and PPG data were measured for a total of 5 min. To minimize the harmful noise level, the volume did not exceed 85 dB, and the provision time was set to 5 min. The volume of aHRSR was (60–65) dB, similar to that of normal conversation, while 75 dB represents the maximum volume produced in this study. Since the ANS can change in many ways depending on the condition of the test subject, the experiment was conducted within 1 h to minimize this in the set environment. Data were measured in the same time band reflecting the circadian system drives (Scheer et al., 2010).

### 2.4 Data calculation and analysis

It is important to detect peak points in the PPG data, which indicate the maximum contraction point of the heart to obtain HRV data. We were able to obtain the RR interval data by calculating the time interval between the peaks detected through Matlab's “findpeaks” function. RR interval data were utilized to obtain HRV, SDNN, and RMSSD. In addition, the power density spectrum (PSD) of the LF and HF regions was calculated using Matlab's “bandpower” function to confirm the activity of the ANS.

To observe real‐time changes, SDNN and RMSSD were used to analyze the RR interval data to identify cardiovascular changes in the time domain (Equations [1] and [2]). Each indicator was divided into 15 s increments and divided into 20 sections in the sitting and standing posture, respectively, with a total of 40 sections. Additionally, the values were standardized to clarify the pattern of changes according to the stimulation. To observe physiological changes in the ANS, HRV per 5 min was transformed into the frequency domain using the Fast Fourier transform to confirm the changes of ANS. The converted HRV was divided into LF in the range (0.04–0.15) Hz and HF in the range (0.15–0.4) Hz using Matlab's filter function (Burr, 2007; Von Rosenberg et al., 2017), and the PSD for each area was calculated (Equations [3] and [4]). All data were to compare the difference between the non‐stimulated state and the stimulated state in the same motion according to the alternative hypothesis, so the aHRSR results were analyzed using the two‐tail test to compare the change in indicators before and after stimulation.

(1) $$\text{SDNN}=\sqrt{\frac{1}{n}\sum_{i=1}^{n}{\left({\mathrm{RR}}_{i}-{\mathrm{RR}}_{\text{mean}}\right)}^{2}}$$

(2) $$\text{RMSSD}=\sqrt{\frac{1}{n-1}\sum_{i=1}^{n-1}{\left({\mathrm{RR}}_{i+1}-{\mathrm{RR}}_{i}\right)}^{2}}$$

(3) $$\mathrm{LF}\left(\mathrm{nu}\right)=\frac{\mathrm{LF}}{\mathrm{LF}+\mathrm{HF}}$$

(4) $$\mathrm{HF}\left(\mathrm{nu}\right)=\frac{\mathrm{HF}}{\mathrm{LF}+\mathrm{HF}}$$

## 3 RESULTS

### 3.1 Effects of sound stimulation on dynamic movement

**FIGURE 3:**
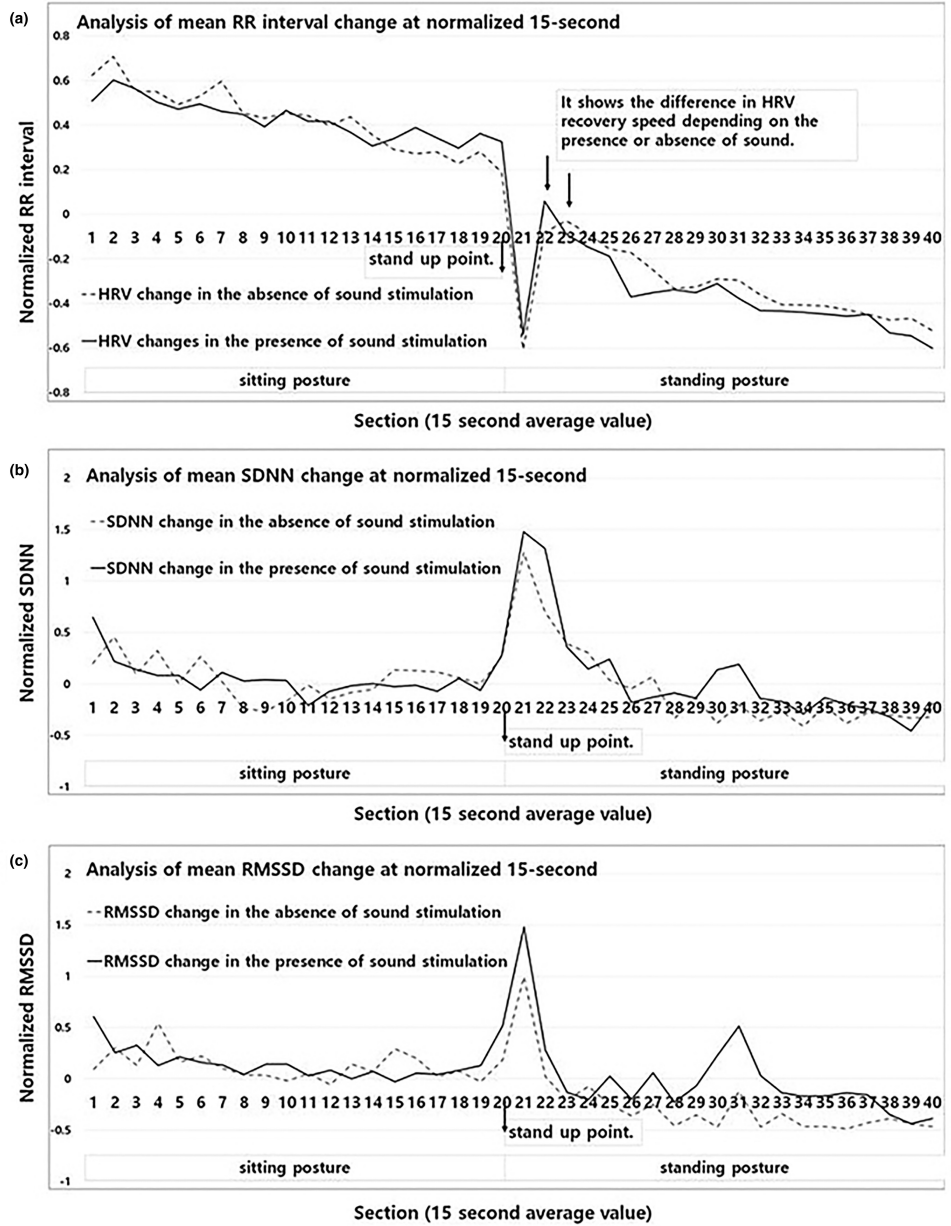
Real‐time RR interval, the standard deviation of the NN intervals (SDNN), the square root of the mean squared differences of successive NN intervals (RMSSD) change pattern. (a) Real‐time changes in the cardiovascular system, (b) the SDNN shows changes in the sympathetic nervous system, (c) the RMSSD shows changes in the parasympathetic nervous system.

After the minimum point of the RR interval that occurs in the process of change, the increase and change patterns of the RR interval were observed to measure the recovery time according to the abrupt change in the autonomic nervous system. The time domain indicators of real‐time RR interval, SDNN, and RMSSD show different patterns of change. The change from the real‐time RR interval to the standing posture shows a sharp decrease in the RR interval, regardless of the presence or absence of aHRSR (section 21 of Figure 3a). This area is the position where the change in potential energy of the cardiovascular system is applied, and the blood pressure is reduced. Accordingly, the human body activates the SNS to increase blood pressure, and the PNS is activated by the feedback action of the increased SNS (Table 1). This is confirmed by the rapid decrease of RR and the rapid increase of SDNN and RMSSD that are shown in Figure 3.

**TABLE 1:** Time domain average indicator values per section after change in posture.

	Section				
	21	22	23	24	25
RR interval without aHRSR (s)	0.627 ± 0.07	0.69 ± 0.11	0.697 ± 0.11	0.69 ± 0.11	0.68 ± 0.11
RR interval with aHRSR (s)	0.665 ± 0.08	0.72 ± 0.12	0.70 ± 0.11	0.70 ± 0.11	0.69 ± 0.11
SDNN without aHRSR (ms)	85 ± 49	67 ± 38	58 ± 39	55 ± 46	47 ± 37
SDNN with aHRSR (ms)	91 ± 58	86 ± 48	57 ± 32	50 ± 26	53 ± 36
RMSSD without aHRSR (ms)	62 ± 64	32 ± 25	25 ± 16	29 ± 35	24 ± 20
RMSSD with aHRSR (ms)	77 ± 89	40 ± 20	28 ± 21	25 ± 16	32 ± 42

*Note*: The RR interval shown in gray shade represents the maximum recovery value. The values recover faster in the case of a HRSR stimulation than in the case without it.

After the rapid change, the ANS has to normalize blood flow. The recovery time of the RR interval after the maximum reduction (section 21) had a difference of about 15 s with section 22 in the presence of a sound stimulus and section 23 in the absence of aHRSR. That is, aHRSR restored the heart rate at a faster rate. In the recovery phase, SDNN and RMSSD maintained higher values during sound stimulation for sections 21–22 (Figure 3b,c). Additionally, as shown in Figure 3c, the base line of RMSSD significantly increased during sound stimulation of aHRSR (sections 24–37). These changes indicate that aHRSR stimulation both affects the instantaneous changes in the ANS and continuously affects the ANS.

### 3.2 Change stability of the autonomic nervous system

**FIGURE 4:**
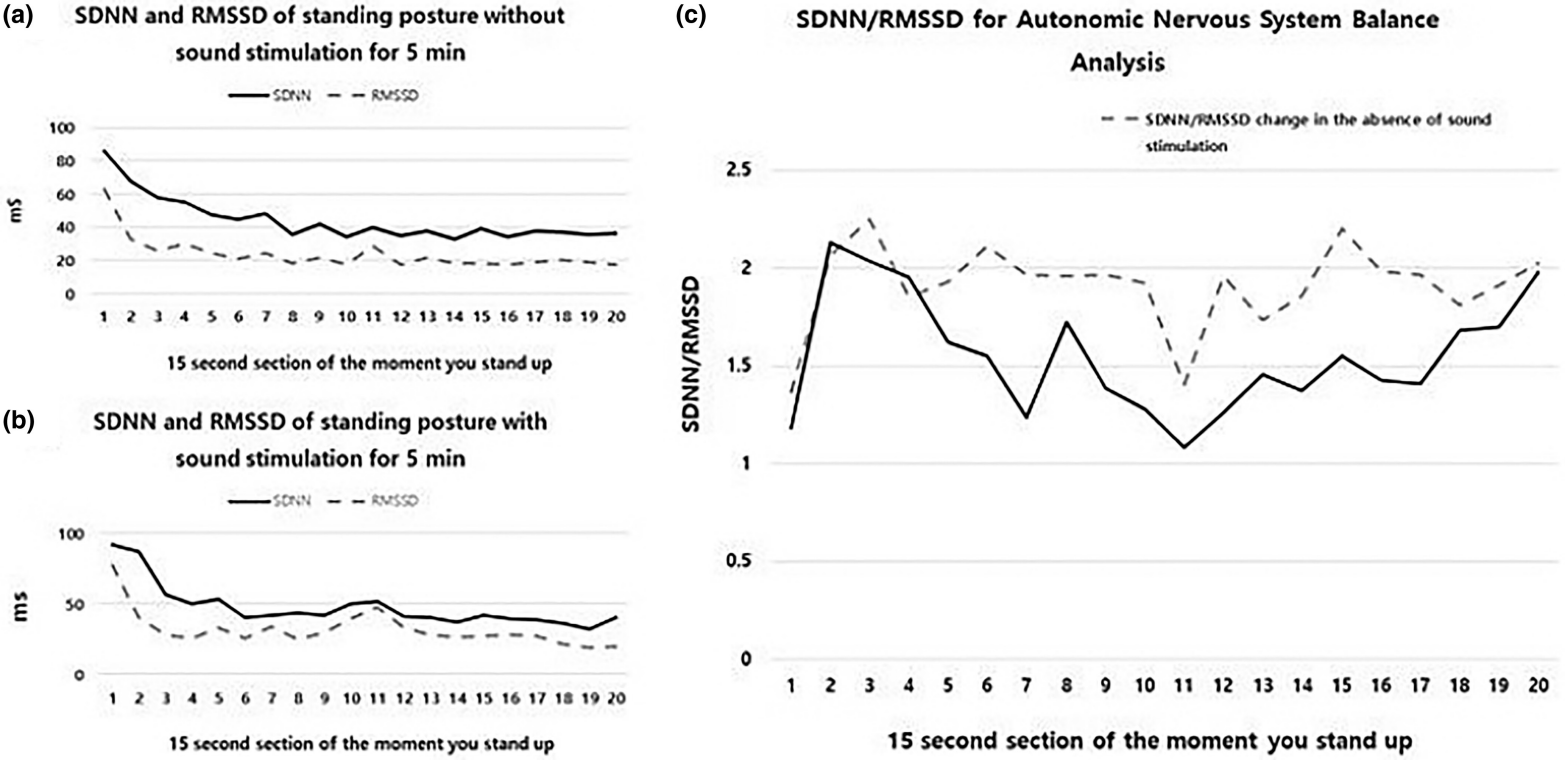
SDNN and RMSSD values and SDNN/RMSSD ratio in the standing position, with or without the average heart rate sound resonance.

SDNN representing the total ANS and SNS, and RMSSD representing the PNS activity were compared according to the presence or absence of aHRSR. After standing up, the mean SDNN was (44.16 ± 13.11) ms and RMSSD (23.73 ± 9.95) ms without aHRSR, and the mean SDNN was (47.85 ± 15.16) ms and RMSSD (31.89 ± 12.48) ms with aHRSR. As shown in Figure 4a,b, the mean values of SDNN and RMSSD increased, but among them, RMSSD increased significantly (*p* < 0.05). Additionally, these results show that the SDNN/RMSSD (Figure 4c), which indicates the balance of the ANS, decreases from an average of (1.91–1.55) (*p* < 0.05). The results show that aHRSR induces overall ANS activity, but a large portion of it induces PNS activity.

### 3.3 Change homeostasis of the autonomic nervous system

Finally, the rate of change in the PSD values of the frequency domain, which occurred with or without aHRSR, was analyzed (Table 2). In using aHRSR, the change total rate of the LF, HF, and LF/HF values decreased significantly (*p* < 0.05). In the absence of aHRSR stimulation, a significant change was shown according to the change in posture (*p* < 0.05); but when aHRSR was stimulated, a significant change was not show (*p* > 0.05). These results show that aHRSR reduces ANS changes, and enhances stability and homeostasis.

**TABLE 2:** Changes in LF, HF, and LF/HF values that appear when the posture changes according to the average heart rate sound resonance.

	Without aHRSR			With aHRSR		
	Phase 2 (Mean ± SD)	Phase 3 (Mean ± SD)	Rate of change (%)	Phase 2 (Mean ± SD)	Phase 3 (Mean ± SD)	Rate of change (%)
LF ($V^{2}/\mathrm{Hz}$)	45.80 ± 20.26	40.23 ± 16.38	−13.84 (*p* < 0.05)	46.54 ± 21.48	42.76 ± 17.78	−8.83 (*p* > 0.05)
HF ($V^{2}/\mathrm{Hz}$)	54.39 ± 19.06	60.84 ± 14.51	10.59 (*p* < 0.05)	55.70 ± 20.84	57.58 ± 16.06	3.27 (*p* > 0.05)
LF/HF	0.84	0.66	−21.42	0.83	0.74	−10.84

## 4 DISCUSSION

The aHRSR proposed in this paper is a method that is used to minimize instantaneous ANS imbalances and changes that may occur in normal individuals. Accordingly, we tried to analyze the stability of the property of maintaining a constant state, and the homeostasis of the property of restoring the state to its original state in response to various stimulation. The increase and decrease of SDNN and RMSSD have various patterns of change depending on lifestyle, age, and disease. As a result of analyzing the SDNN and RMSSD of a total of 3483 adults aged (18–65) years in Korea, the averages were ([39.6 ± 22.1] and [29.7 ± 18.1]) ms, respectively. However, as age increases, or when smoking or drinking alcohol, SDNN and RMSSD tend to decrease significantly; in contrast, according to the frequency of exercise, it shows a tendency to increase (Kim & Woo, 2011). In terms of disease, it was significantly decreased in schizophrenia, bipolar disorder, and cardiovascular disease (Moon et al., 2013). These changes increase with the stable activity and tension of the ANS (Routledge et al., 2010), and decrease with the instability of the ANS (Murata et al., 1994). That is, the average ms size observation of SDNN and RMSSD can confirm the stability and instability of the ANS.

In this paper, the effects of aHRSR on dynamic movement were analyzed for the first time. The aHRSR showed a fast RR interval recovery time of about 15 s. In other words, changes in the ANS caused by dynamic movement were quickly stabilized. Figure 4b,c reveals that sections 20–23 show similar change patterns with and without aHRSR, but in Figure 4b, SDNN maintained an uptrend during aHRSR in sections 22–23. In this study, this cause was judged as a response to a stimulation. The moment the subject stands up, aHRSR induces the activity of SNS by providing a new input variable called auditory stimulus (Lee et al., 2010). As a result, it is judged that aHRSR maintained overall ANS activity. However, the sensory adaptive system decreases its responsiveness to constant or identical stimulation (O'Mahony, 1986; Pearson, 2000; Weill‐Duflos et al., 2020). For this reason, the sensory adaptation time of aHRSR is 45 s, and the PNS is activated along with the SNS as a momentary reaction; and as the SNS adapts, the PNS reaction effect seems to be continuously induced.

In this study, the change to the standing posture greatly increased the activity and tension of the ANS. Importantly, aHRSR increased the mean values of SDNN and RMSSD from ([44.16 ± 13.11] to [47.85 ± 15.16]) ms, and from ([23.73 ± 9.95] to [31.89 ± 12.48]) ms, respectively. That is, these results show that aHRSR stimulation can increase the stability of the ANS and reduce the instability, based on the results of previous studies. Next, to confirm the effect of aHRSR on the homeostasis of the ANS, the rate of change of LF and HF according to the presence or absence of aHRSR was checked. In all indicators of LF, HF, and LF/HF, the rate of change was decreased upon stimulation with aHRSR. These results indicate that stimulation of aHRSR enhances resistance to changes in the ANS. That is, it is judged to help maintain homeostasis. Then, to confirm the effect of aHRSR on the homeostasis of the ANS, the rate of change of LF and HF according to the presence or absence of aHRSR was checked. In all indicators of LF, HF, and LF/HF, the rate of change was decreased upon stimulation with aHRSR. The difference between LF and HF values during posture change was (−5.57 and + 6.45), but changed to (−3.78 and + 1.88) according to aHRSR. These results indicate that stimulation of aHRSR enhances resistance to changes in the ANS. That is, it is judged to help maintain homeostasis.

The reason for the judgment is that aHRSR stimulation stimulates the vagus nerve connected to the brain stem. The vagus nerve is an important component of the parasympathetic nerve, and is involved in motor control and heart rate control. Noninvasive vagus nerve stimulation (Clancy et al., 2014) exists as a technique to stimulate the vagus nerve, and its effect significantly reduced the ratio of SDNN/RMSSD, and led to changes in the ANS toward the vagus nerve dominance. The results show the same pattern as in this study (Burr, 2007; Clancy et al., 2014; DeGiorgio et al., 2010; Geng, Liu, et al., 2022; Geng, Yang, et al., 2022; Kim & Woo, 2011; Lee et al., 2010; Moon et al., 2013; Murata et al., 1994; O'Mahony, 1986; Pearson, 2000; Routledge et al., 2010; Weill‐Duflos et al., 2020). The results of this study confirm that the relative size of RMSSD is higher than SDNN, as shown in Figure 4c. This indicates that sound stimulation that mimics an individual's heart rate like aHRSR can affect the vagus nerve (Kim et al., 2020, 2021; Park et al., 2015; Wong & Figueroa, 2019). Also, one of the important factors is that the potential of vagus nerve stimulation methods combined with slow breathing is considered (Szulczewski, 2022).

Finally, based on these contents, this study was able to confirm important points. These are the cardiac baroreflex sensitivity (BRS) and blood pressure (BP). Cardiac baroreflex plays an important role in thermodynamic stability and cardioprotection by modulating vascular resistance. However, the delay time of the feedback control system that adjusts the BP is about 10 s. In the movements presented in this study, stimulation of the aHRSR accelerated maximal HRV recovery by 15 s. Additionally, research papers suggest that RMSSD may reflect BRS. The vagus nerve also carries information about every blood pressure beat to the brainstem. In other words, the magnitude of RMSSD enhanced by aHRSR enhanced BRS, suggesting that it may be involved in BP in the short‐ and long‐term. Based on this, it is suggested that stimulation of aHRSR both improves the stability and homeostasis of the autonomic nervous system, and induces changes in the human body through additional physiological stimulation (Savabi et al., 2020; Skow et al., 2022; Swenne, 2013). This shows various possibilities for application, such as healthcare methods and biofeedback methods that individuals can use (Kim et al., 2023).

This study has two limitations. Although the study was conducted in controlled environments and conditions, not all of the ANS was controlled. The ANS connects organs and spreads throughout the body, and has both efferent and afferent neural signaling systems. Therefore, changes in the condition of other organs can cause changes in the heart. That is, even if the external environment is controlled, it is not possible to match the sleep time, food intake, smoking history, age, and physical condition that could change the ANS activity in the human body. However, in this study, to solve this problem, the circadian system drives were matched, and the ANS that could be changed was minimized by measuring in the same time band as the experimental procedure within 1 h per subject. The other limitation was that the results of this study did not identify any clinical or physiological mechanisms in humans. In this study, the effect of aHRSR was established by using the existing mechanism, and then, the effect of aHRSR was confirmed by analyzing the change in the HRV index obtained through the results. However, the result could only confirm the final result of the body of aHRSR stimulation, and the exact mechanism could not be identified. To secure these shortcomings, the same change pattern was found based on various existing studies, and the credibility of the aHRSR stimulation was secured by writing an interpretation and review suitable for the change.

## 5 CONCLUSION

In this study, we proposed aHRSR, a new method for improving ANS stability and homeostasis that can be used by the general public without disease. It was confirmed that aHRSR can affect the ANS at a much safer level than electrical and chemical stimulation, which can cause side effects and complications. The function of aHRST induces ANS activity and increases RMSSD, an indicator of the PNS, by assisting respiration through stimulation with cycles. Through this, it is expected that this study will develop into a new technology that can prevent central nervous system diseases by suggesting a new development direction for the biofeedback method using individual biosignals.

## AUTHOR CONTRIBUTIONS

DCK and YJL designed the experimental method to secure the composition and clinical value of this study. DKC and NHK measured the data using the designed process and method, and conducted data analysis of the participants. SMK and JYK discussed the cause and reason of the analyzed result with DCK, and finally suggested the effect and meaning of sound stimulation. SMK and JYK reviewed and edited the manuscript, and approved the final version of the manuscript.

## FUNDING INFORMATION

This research was supported by a grant RS‐2023‐00215716 from Ministry of Food and Drug Safety in 2023

## CONFLICT OF INTEREST STATEMENT

The authors declare that they have no conflict of interest.

## ETHICS STATEMENT

Study approval statement: The study was conducted according to the guidelines of the Declaration of Helsinki, and approved by the Institutional Review Board or Ethics Committee of the Dongguk University Institutional Review Board (DUIRB‐202109‐12).

Consent to participate statement: Informed consent was obtained from all participants involved in the study.

## Data Availability

The data are available upon reasonable request. The data should be requested by email to the corresponding author, with a clear reason provided for the purpose of use. If the request is reasonable, we will forward the relevant data after contacting the consenter according to the contents of the consent.
